# Retiring Address

**Published:** 1874-01

**Authors:** L. Buffett

**Affiliations:** President of the Ohio State Dental Society


### RETIRING ADDRESS.

BY L. BUFFETT, PRESIDENT OF THE OHIO STATE DENTAL SOCIETY, DEC. 5, 1873.

Gentlemen : At the close of this, the eighth session of the Ohio State Dental Society, it may be well to take a cursory view of these few years, and see what progress has been made; the benefits we have derived ; the influence it has had upon others of the profession, in this state, although not connected with it ; the lessons to be learned.

When first organized, it had but a few members ; some of whom were earnest workers, willing to give their time and talent, not only to this society, but were workers for the good of the whole profession. Gradually it has increased, until now it has about one hundred and twenty-five members in good standing. A number have become disconnected with the Society, mostly by negligence in not conforming to the payment of dues and non-attendance.

Our ranks have been but little broken by death. In the last year, we have have lost our Vice-President, Dr. B. F. Rosson. His labors are done and he knows of that we know not of, the other world. We shall miss him—will he miss us?

But few complaints have been made to the Society for non-professional conduct of its members. As a whole, harmony has prevailed, and with pleasant recollections we can look back to each annual meeting.

Intellectually it has been of great advantage. New ideas have been presented that we would have received in no other way. To some it has been the first school for them to enter, and we have all been teachers, and have all been scholars ; and if the teachers have been a little cross at times, and the scholars a little stubborn and unruly at times, it has only been a part of the discipline bringing us more in harmony. And we to-day are better men, better dentists for the Ohio State Dental Society.

Socially we have gained much ; we know each other better. The good we have found, I am sure, more than ten times counter-balances the evil. New friends have been added to our list ; some that we know will stand by us in dark days, if they should ever come, and come they will to some of us.

The position that this Society has taken to elevate the profession has brought almost every dentist in this State to feel that to practice without proper qualifications is committing a moral wrong, and that they should receive the condemnation of the profession and of the community in which they practice.

I don't wish to be understood to say that all those that have not met the requirements of the law are thus to be condemned ; for we know some are well qualified. But we do know that the majority of the opposers of the law, as it was, are the dead-weights not given to the advancement of the profession or of themselves intellectually.

Notwithstanding the opposition that the law received, it has done much good. More reading has been accomplished by the dentists of this State in the last five years than in any ten years prior to the passage of the Dental Law. Truly may this Society feel proud of accomplishing what it has, even if the results are not all that we could wish.

Much still remains to be done. The field is large, and the workers for dollars and cents are large ; but the workers for science and advancement of others, easily numbered. If every member of this body were in earnest, doing all that was for them to do, great would be the results ; the standard of excellence would soon be raised to a much higher degree of perfection than at present. And we might, in truth, be all that we claim—a scientific body.

As dentists, we should consider the influence we have upon each other, not only in our Society gatherings, but in private intercourse and indirectly through patients.

Let our practice be in harmony with our public professions. Let it not be said of us that we condemn here and at other gatherings those things we go back to our offices and practice. Let no misstatements, no deception be made, in any way, to sustain pet theories, or to appear greater than we ought. Rest assured we will all find our proper level; and others know it sooner than we do ourselves.

Selfishness and deceit are the besetting sins of too many of us. I know we do not like to own it, much less to be told of it. We look upon ourselves through a mirror that flatters; and, if that is not sufficient, some friend tells us the mirror does not do us justice, and then deceit takes from us much of the good it is ours to possess.

It is the duty of every one to render just dues to his fellow practitioners, acknowledging the benefits we have derived from their labors and investigations. In no way rob them by claiming to be the originators of that which they have so freely given. We well know that those who have done the most for the profession have not made money thereby. Others reap the benefits financially; and, if the time should come when they should need, let not the hand be stayed from giving—no, I would not say giving, but paying our indebtedness; for some at least of that we possess, by right, belongs to them. They taught us what and how to do, and shall no recompense be given?

In looking over the past, we find many sins of omission as well as commission. We have omitted to improve ourselves as we ought; to treat our professional brother with that respect that is due; the societies have not received enough of our time and thought; dental colleges, the support that is needed; for it is to them that the future is depending for educated dentists, and their prosperity is in the hands of the profession. Rest assured they will be all we are willing to make them, having a high or low standard of excellence.

Gentlemen, the past has been productive of much good to us, as individual members and as a Society. Let us by careful reading and investigation make the futnre yield us a greater richer harvest. We must labor. The lazy man has no business to be a dentist ; he will probably be one only in name.

The great problems of this life are not how we can avoid the difficulties and responsibilities, but how to overcome them ; how to keep mind and body in such harmony, in such a healthy working condition, that we do not shrink from assuming those duties that are laid upon us.

In conclusion, I would say : Let us be true to ourselves, honest to our patients, just to fellow practitioners, and labor in love for the good of the whole profession.

